# Cold, dry air is associated with influenza and pneumonia mortality in Auckland, New Zealand

**DOI:** 10.1111/irv.12369

**Published:** 2016-05-17

**Authors:** Robert E. Davis, Erin Dougherty, Colin McArthur, Qiu Sue Huang, Michael G. Baker

**Affiliations:** ^1^ Department of Environmental SciencesUniversity of VirginiaCharlottesvilleVAUSA; ^2^ Auckland City HospitalAucklandNew Zealand; ^3^ Institute of Environmental Science and ResearchWellingtonNew Zealand; ^4^ University of Otago‐WellingtonWellingtonNew Zealand

**Keywords:** Air temperature, climate, humidity, influenza mortality, seasonality, weather

## Abstract

The relationship between weather and influenza and pneumonia mortality was examined retrospectively using daily data from 1980 to 2009 in Auckland, New Zealand, a humid, subtropical location. Mortality events, defined when mortality exceeded 0·95 standard deviation above the mean, followed periods of anomalously cold air (*t*_a.m._ = −4·1, *P* < 0·01; *t*_p.m._ = −4·2, *P* < 0·01) and/or anomalously dry air (*t*_a.m._ = −4·1, *P* < 0·01; *t*_p.m._ = −3·8, *P* < 0·01) by up to 19 days. These results suggest that respiratory infection is enhanced during unusually cold conditions and during conditions with unusually low humidity, even in a subtropical location where humidity is typically high.

## Introduction

The strong seasonality evident in mortality records from mid‐latitude locations suggests that climatic factors may amplify cold‐season death rates.^1^, ^2^ As respiratory mortality exhibits the strongest winter peak of all broad mortality categories,^3^ it has been suggested that influenza and related mortalities may (significantly or perhaps even entirely) account for the winter mortality peak.^1^

Reasons for the seasonal pattern in influenza mortality remain unclear. Recent research suggests a connection to weather, particularly cold and/or dry (low humidity) air.^3^ Possible explanations for this relationship includes factors related to virus characteristics,^4^, ^5^ drying of nasal mucous membranes,^6^ enhanced airborne transmission,^7^ and human behavioral factors.^8^

Several studies in the United States have linked the timing^9^ and severity^10^ of influenza mortality peaks to periods of cold and/or dry air, in which the onset of the mortality event followed the dry period by about three weeks, thereby accounting for a disease latency period. Because influenza peaks in the cold season in both the Northern Hemisphere and Southern Hemisphere,^11^ we chose to test this cold, dry air hypothesis in a Southern Hemisphere, mid‐latitude location. Auckland, New Zealand, provides an interesting comparison site because of its geographical and climatic situation. Auckland, on the northern island of New Zealand, has a maritime climate and the nearest source of dry air (Australia) is remote, so low humidity air masses are rare. Thus, Auckland provides an interesting test case in which to examine the purported linkage between influenza and cold and dry weather.

## Data and methods

Daily mortality counts of pneumonia and influenza (P&I) were tallied from 1980 to 2009 from the Mortality Collection managed by the New Zealand Ministry of Health. Pneumonia and influenza are commonly grouped in retrospective analyses as it is often difficult to ascribe a given mortality event to influenza because of the lack of laboratory confirmation and because pneumonia deaths may occur when the influenza virus is no longer detectable.^12^ As our study period spans two different coding periods of the International Classification of Diseases (ICD), we used the 9th revision (ICD‐9) P&I codes 480–488 prior to January 1, 2000, and the 10th revision (ICD‐10) codes J09–J18 afterward. Mortality counts were determined based on the decedent's residence in the Auckland metropolitan area based on definitions from the 2006 Census Area list.

**Figure 1:**
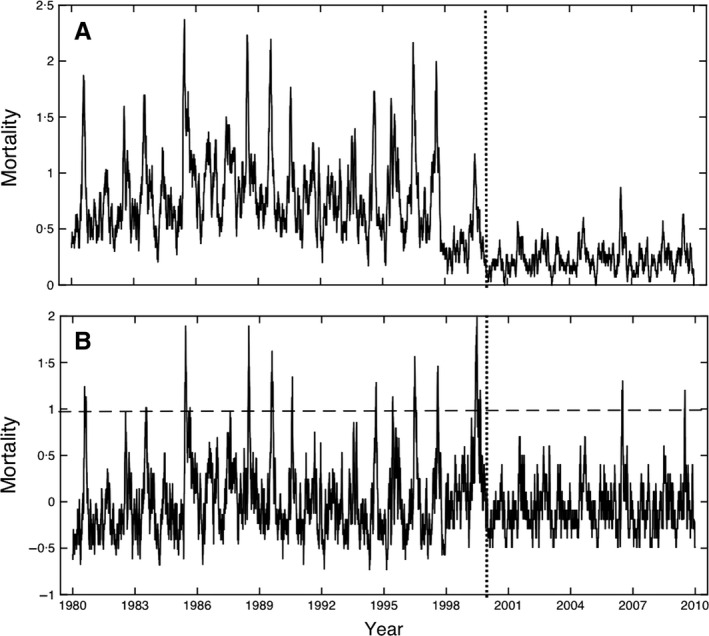
(A) Daily pneumonia and influenza mortality time series for Auckland, New Zealand, from 1980 to 2009. A 19‐day leading average smoother has been applied to these data. Prior to the conversion from ICD‐9 to ICD‐10 coding in 2000 (the dotted vertical line), declines in the mean and variance are evident. (B) Revised time series after *z*‐scoring by ICD period. Mortality “episodes” include three or more consecutive days with *z* ≥ +0·95 (the dashed horizontal line). Please see text for details.

The raw mortality time series exhibits significant seasonality and a decline in both mean and variance beginning in 1998 prior to the ICD‐9 to ICD‐10 conversion (Figure 1A). To address this discontinuity, each day's mortality count was converted to a *z*‐score by subtracting the mean and dividing by the standard deviation of each period (1980–1998 and 1999–2009). To account for the latency period between infection and death, a 19‐day leading average smoother was applied to the *z*‐scored time series. This smoother was selected based upon prior research^9^, ^10^ and after testing several different filter types and lengths.

The resulting P&I mortality frequency histogram (see Supporting Information) exhibited a long, positive tail starting at *z* = +0·95, so this value was chosen as a threshold to identify high mortality “events,” or days with high mortality (*N* = 303 days). Longer mortality “episodes” were defined as three or more consecutive events with no intervening period of more than five non‐event days—13 such episodes were defined, and these varied from 7‐ to 50‐day duration (Figure 1B and Table 1). By using this threshold approach to identify high mortality days, we hope to effectively isolate the events and episodes that are most likely to be linked to P&I with fewer confounding effects from other causes.

**Table 1:** Characteristics of P&I mortality episodes

Episode #	Start date	End date	Duration	Total mortality	Mean daily mortality
1	July 26, 1980	August 24, 1980	30	30·72	1·02
2	May 27, 1985	June 28, 1985	33	45·35	1·37
3	June 14, 1988	July 11, 1988	28	39·81	1·42
4	July 26, 1989	August 28, 1989	34	42·69	1·26
5	July 23, 1990	August 5, 1990	14	15·11	1·08
6	August 7, 1994	August 19, 1994	13	14·36	1·10
7	May 30, 1995	June 8, 1995	10	10·31	1·03
8	June 14, 1996	July 17, 1996	34	40·61	1·19
9	July 27, 1997	August 16, 1997	21	26·36	1·26
10	May 26, 1999	July 14, 1999	50	70·55	1·36
11	August 22, 1999	August 31, 1999	10	10·78	1·08
12	June 26, 2006	July 10, 2006	19	21·66	1·14
13	June 30, 2009	July 6, 2009	7	7·59	1·08

Weather data were acquired for Auckland International Airport (station #1962) from National Institute of Water and Atmospheric Research archives. Data analyzed included morning and afternoon air temperature (*T*) and dew point temperature (*T*_d_), the latter of which is a measure of atmospheric humidity used in prior influenza research.^10^ Observation times for morning (0500 or 0600 Local Standard Time (LST)) and afternoon (1200 or 1400 LST) varied over the course of the year based on the local times when 3‐hourly observations were recorded. To remove seasonality, these data were converted to *z*‐scores using the mean and standard deviation for each day of the year and were subsequently smoothed with a 3‐day lagging moving average filter. For the weather data, this short filter length was chosen to reflect the high daily variability inherent in Auckland's weather.

The weather on P&I event and episode days was compared to all other days using a one‐sample *t*‐test, with the alternative hypothesis that mortality would be elevated on colder and drier days (assuming a 19‐day lag). Because the data have been *z*‐scored to a zero mean, the *t*‐test is comparing temperature and dew point departures from a zero baseline (*P* ≤ 0·01). Conversely, we examined P&I mortality on unusually cold days and on days with low humidity. Temporal autocorrelation and potential inflation of the true degrees of freedom was addressed by altering the effective sample size (see Supporting Information).

## Results

P&I mortality is significantly elevated about three weeks after both cold days and dry days (Table 2). Relationships are slightly stronger with temperature than with dew point temperature, but as these variables are positively correlated, similar responses are expected. There likewise are associations between cold and dry air and extended mortality episodes (Table 2). Because the disease latency is built into the analysis, these results indicate that below normal temperature and humidity precede broad peaks in P&I mortality by up to 19 days. In addition, days with elevated mortality tend to be colder and drier (after accounting for lagged effects) (Table 2).

**Table 2:** Results from one‐sample *t*‐tests (A) for individual high P&I mortality events and high mortality episodes and (B) for P&I mortality events on unusually cold or dry days

(A)						
	Event			Episode		
	Mean	SD	*t*	Mean	SD	*t*
T^c^ (a.m.)^d^	−1·01^b^	0·74	−4·05	−1·01^b^	0·74	−4·05
T (p.m.)^e^	−1·11^b^	0·51	−4·20	−1·11^b^	0·50	−4·28
T_d_ f (a.m.)	−0·92^b^	0·73	−4·09	−0·93^b^	0·73	−4·13
T_d_ (p.m.)	−0·78^b^	0·72	−3·74	−0·80^b^	0·72	−3·83

(B)			
	Mean	SD	*t*
Mortality^g^ [T(a.m.) ≤ 1]	0·27^a^	0·44	2·36
Mortality [T (p.m.) ≤ 1]	0·31^a^	0·43	2·95
Mortality [T_d_(a.m.) ≤ 1]	0·27^a^	0·43	2·30
Mortality [T_d_ (p.m.) ≤ 1]	0·24^a^	0·43	2·00

a *P* < 0·05.

b *P* < 0·01.

c Air temperature *z*‐score (°C).

d 0500 or 0600 LST.

e 1200 or 1400 LST.

f Dew point temperature *z*‐score (°C).

g P&I mortality *z*‐score.

The weather data have been standardized to remove seasonality. Therefore, our results indicate that *anomalously* cold and dry days (relative to the mean for that day of the year) are associated with elevated P&I mortality. Influenza has a very pronounced cold‐season peak (from June to October) in Auckland, where winters tend to be moderately cool and humid (Table 3). Similar results were found in a comparable study for New York City,^10^ where winters are both colder and significantly drier (Table 3). Thus, analogous relationships were found in Auckland *despite the general lack of dry (low dew point) air* in this subtropical island location. Unusually cold and dry periods may be associated with conditions that enhance transmission and survival of the influenza virus or other respiratory pathogens, but the atmospheric triggers appear to depend upon relative departures rather than absolute temperature and humidity conditions.

**Table 3:** Average maximum and minimum monthly air temperature and dew point temperature during the main influenza season for Auckland (June–October) and New York City (November–March)

	AKL^a^		NYC^b^		AKL		NYC	
	*T*_max_ c	*T*_min_	*T*_max_	*T*_min_	*T*_d__max_ d	*T*_d min_	*T*_d max_	*T*_d min_
June/November	15·0	8·9	11·7	4·4	11·7	6·7	5·0	−2·2
July/December	13·9	7·8	6·1	0·0	10·6	6·1	0·0	−8·3
August/January	14·4	8·3	3·3	−4·4	10·0	6·1	−2·2	−11·7
September/February	16·1	9·4	4·4	−2·8	11·7	7·2	−1·7	−10·6
October/March	17·2	11·1	8·9	0	12·2	8·3	1·7	−6·7

a Auckland, New Zealand.

b New York City, USA.

c Air temperature, measured in °C.

d Dew point temperature, measured in °C.

Indoor heating (without humidification) may lengthen survival times of respiratory viruses and weaken nasal defense mechanisms. Although central heating is not common in Auckland, indoor heating will be used most often during abnormally cool periods in winter when respiratory infection is most prevalent, although fuel poverty is an important problem for deprived groups in New Zealand.^13^ People tend to gather in closer proximity during cold periods, enhancing the likelihood of both airborne and contact transmission. It is likely that the reasons for a pneumonina and influenza/climate link are multifaceted and related to the virus itself, the host, as well as to behavioral factors.

As this was a correlative study based on long‐term data archives, several limitations must be considered. New disease coding rules introduced with ICD‐10 reduced the use of pneumonia codes as the underlying cause of death in those cases with other serious chronic illnesses, thereby impacting the time series around the time of the coding transition in 1998–2000. Combining pneumonia and influenza mortality into a single group, while a necessity without laboratory confirmation of influenza, means that the true impact of the influenza virus is unknown (estimates are that influenza accounts for 1·6% of all medical deaths in New Zealand).^14^ Although we had access to data on the dominant seasonal influenza strain, these data were only available for a portion of the study period and the results were inconclusive and internally inconsistent based upon the limited sample size. Additional research is needed on the relation between weather and specific respiratory pathogens, and these results on daily weather and pneumonia and influenza mortality need to be validated in other settings. Because air and dew point temperature are highly correlated in Auckland, they are confounding variables and it is difficult to clearly identify which is more important to P&I mortality. These results show a slightly stronger influence of low temperatures (see Supporting Information), but this topic requires additional research.

## Conclusions

Abnormally cold, dry air tends to precede days and periods with high pneumonia and influenza mortality in Auckland, New Zealand, by up to three weeks. Given the ability to forecast cold and dry periods in advance, such information could potentially be used for “real‐time” respiratory infection forecasting. This could help reduce the impact of pneumonia and influenza in a number of ways: communication to vulnerable groups (particularly the elderly and those with established respiratory disease), deployment of preventive measures (e.g., vaccine and antivirals), and planning for increased service demands in hospitals. Before doing this, it would be useful to review the various approaches to forecasting, and to calibrate and validate the forecast models.^15^

## Supporting information

Figure S1. Frequency histogram of smoothed and *z*‐scored P&I mortality in Auckland, New Zealand, 1980–2009.
Table S1. Summary of linear regression results when the second variable is fitted to the residuals of the regression on the first variable.
