# Supplementary material for: Cold, dry air is associated with influenza and pneumonia mortality in Auckland, New Zealand
Source: Influenza Other Respir Viruses. 2016 May 17;10(4):310–3. doi: 10.1111/irv.12369 (PMC4910181; doi:10.1111/irv.12369)
Supplement: Supplementary file 1 — Figure S1. Frequency histogram of smoothed and z‐scored P&I mortality in Auckland, New Zealand, 1980–2009. Table S1. Summary of linear regression results when the second variable is fitted to the residuals of the regression on the first variable. [file IRV-10-310-s001.docx]

**Supplementary Materials**

1. The frequency histogram exhibits a long, positive tail of days with high P&I mortality (Figure S1). We selected a threshold of +0.95 to identify high mortality days and episodes.


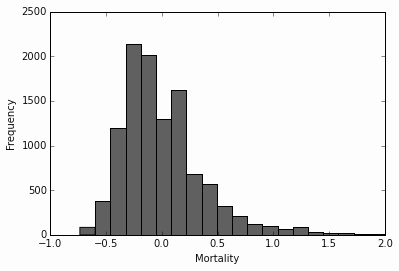


**Figure S1**. Frequency histogram of smoothed and z-scored P&I mortality in Auckland, New Zealand, 1980–2009.

2. Time series data for certain weather variables often exhibit high serial correlation and the actual number of independent observations would be overestimated if this autocorrelation is not addressed. We used the correction suggested by Wilks^1^ by adjusting the actual sample size (N) to the effective sample size (N’) as follows:

$$N^{'}=\left[ \frac{1-P}{1+P} \right]N$$

where P is the lag-1 temporal autocorrelation. The effective sample size is then used in the calculation of the test statistic.

3. Results of T vs. T_d_ comparisons.

To determine if low temperature or dry air is more strongly associated with P&I mortality peaks, we employed a regression approach in which we fitted a linear model using one variable (either T or T_d_) and then fitted the residuals using the other. This was repeated for morning and afternoon observations (Table S1).

**Table S1.** Summary of linear regression results when the second variable is fitted to the residuals of the regression on the first variable. This procedure was repeated for morning and afternoon air temperature (T) and dew point temperature (T_d_).

|  | **R^2^** | **F** | **p** | **sign** |
| --- | --- | --- | --- | --- |
| 1) T (a.m.) and mortality* | 0.18 | 2435.0 | 0.00 | – |
| Residuals and T_d_ (a.m.) | 0.00 | 0.02 | 0.90 |  |
| 2) T (p.m.) and mortality* | 0.22 | 3156.3 | 0.00 | – |
| Residuals and T_d_ (p.m.)* | 0.00 | 5.9 | 0.02 | + |
| 3) T_d_ (a.m.) and mortality* | 0.12 | 1514.3 | 0.00 | – |
| Residuals and T (a.m.)* | 0.00 | 49.4 | 0.00 | – |
| 4) T_d_ (p.m.) and mortality* | 0.14 | 1726.7 | 0.00 | – |
| Residuals and T (p.m.)* | 0.03 | 375.2 | 0.00 | – |

* p< 0.05

The linear regressions with temperature are stronger than those with dew point temperature, but afternoon dew point does account for a statistically significant part of the residual variance (morning dew point does not).

When the model is run as a single multiple regression (for morning and afternoon separately or all four variables together) the colinearity between temperature and dew point temperature results in variance inflation and unstable estimates of the regression parameters. Thus, limited evidence suggests a stronger relationship to temperature than moisture, although this topic requires additional investigation.

**Reference**

(1) Wilks DS: Statistical Methods in the Atmospheric Sciences (2^nd^ Edn.). Boston/San Diego/London, Academic Press, 2006.
